# Tissue specificity drives protective immunity against *Staphylococcus aureus* infection

**DOI:** 10.3389/fimmu.2022.795792

**Published:** 2022-08-02

**Authors:** Pavani Beesetty, Youhui Si, Zhaotao Li, Ching Yang, Fan Zhao, Anita S. Chong, Christopher P. Montgomery

**Affiliations:** ^1^ Center for Microbial Pathogenesis, Abigail Wexner Research Institute at Nationwide Children’s Hospital, Columbus, OH, United States; ^2^ Department of Surgery, the University of Chicago, Chicago, IL, United States; ^3^ Department of Veterinary Biosciences, College of Veterinary Medicine, The Ohio State University, Columbus, OH, United States; ^4^ Department of Pediatrics, College of Medicine, The Ohio State University, Columbus, OH, United States; ^5^ Division of Critical Care Medicine, Nationwide Children’s Hospital, Columbus, OH, United States

**Keywords:** MRSA, immunity, skin infection bacteria, pneumonia, antibody, T cells

## Abstract

Infections caused by *Staphylococcus aureus* range from mild to severe and frequently recur. Emerging evidence suggests that the site and severity of infection drive the potency of elicited immune responses and susceptibility to recurrent infection. In this study, we used tractable mouse models of *S. aureus* skin infection (SSTI) and pneumonia to determine the relative magnitude of elicited protective immunity. Surprisingly, despite both SSTI and pneumonia eliciting antibody and local effector T cell responses, only SSTI elicited protective antibody and memory T cell responses and subsequent protection against secondary SSTI and pneumonia. The failure of pneumonia to elicit protective immunity was attributed to an inability of *S. aureus* pneumonia to elicit toxin-specific antibodies that confer protection during secondary infection and was associated with a failure to expand antigen-specific memory T cells. Taken together, these findings emphasize the importance of understanding protective immunity in the context of the tissue-specificity.

## Introduction

*Staphylococcus aureus* infections are common in both health-care and community settings, ranging from mild to severe (1). An epidemic of infections caused by methicillin-resistant *S. aureus* (MRSA) isolates (2) mandates that novel preventative and therapeutic strategies be prioritized. Among these infections, *S. aureus* has emerged as a leading cause of community-associated skin and soft tissue infections (SSTI) and pneumonia (1). Recurrent *S. aureus* infections are common, occurring in at least 50% of otherwise healthy children and adults with SSTI (3, 4). This suggests that *S. aureus* infections do not “naturally” elicit protective immune responses, thereby limiting the ability to exploit understanding of natural responses to guide vaccine development. However, in contrast to SSTI, recurrence among children with invasive *S. aureus* infections appears to be less common (3). This suggests that *S. aureus* infections elicit host immune responses that differ quantitatively and/or qualitatively depending on the primary site of infection. Better understanding the mechanisms by which distinct infectious syndromes elicit immune responses may enable discrimination between populations with different risk of infection and advance vaccine development.

In a mouse model of recurrent *S. aureus* SSTI, we reported that primary SSTI protected BALB/c mice against secondary SSTI *via* both antibody and Th17/IL-17 dependent mechanisms (5, 6). In contrast, C57BL/6 mice were not protected despite robust antibody responses because the immunogenic epitopes within protective antigens cannot bind MHC class II^b^ proteins expressed in this genetic background (6). Recently, Lee et al. reported that *S. aureus* bloodstream infection, but not SSTI, protected C57BL/6 mice against secondary SSTI, suggesting that invasive infection better elicits protective responses (7). However, little is known about the magnitude of immune responses elicited by pneumonia in either preclinical models or humans. We therefore adapted our model of recurrent SSTI to compare local and systemic immune responses following primary SSTI and pneumonia, with the hypothesis that the more invasive infection (i.e. pneumonia) would preferentially elicit protective immunity. Surprisingly, despite eliciting less expansion of local T cells during primary infection, SSTI elicited higher levels of *S. aureus*-specific antibody and more *S. aureus*-specific memory T cells in BALB/c mice, compared with pneumonia. The importance of these responses was demonstrated by the observation that SSTI, but not pneumonia, elicited protection against secondary SSTI and pneumonia.

## Materials and methods

### Mouse models of *S. aureus* infection

Our models of *S. aureus* SSTI and pneumonia have been described (5, 8). All mice used in this study (BALB/c, C57BL/6, or μMT B cell deficient mice on the BALB/c background) were purchased from Taconic or bred at the University of Chicago. The virulence of the USA300 clinical isolate 923 and 923*Δsae* have been described (8, 9). Bacteria were revived from frozen stocks, incubated at 37°C overnight, and subcultured into tryptic soy broth (TSB) and grown at 37°C overnight with shaking (250 rpm). The following morning, the overnight culture was diluted 1:100 in fresh TSB and grown to an OD_600_ of 1.8. The pelleted bacteria were washed in phosphate-buffered saline (PBS), following which they were resuspended in PBS to achieve the desired bacterial density. Prior to inoculation, all mice were sedated with ketamine and xylazine. For SSTI, the flank was shaved and cleaned with ethanol, following which inoculation was performed with 50 μl of the bacterial slurry (1.5 x 10^7^ CFU). For pneumonia, 20 μl of *S. aureus* was instilled by intranasal inoculation. For assessing immune responses following pneumonia, a sublethal inoculum was used (1 x 10^8^ CFU); for assessing protection against lethal pneumonia, a higher inoculum was used (3-4 x 10^8^ CFU). Mice were observed at least twice daily and the severity of illness was quantified using a validated illness severity score (scale 1-6). Mice that were moribund (score of 4 or greater) were sacrificed. For the high inoculum studies, mice were observed for 72 hrs.

Except where noted, secondary infection was performed 6 weeks following the primary infection. For adoptive serum transfer, blood was obtained 4 weeks following secondary infection of BALB/c mice or 2 weeks following vaccination and boost with Al(OH)_3_-adjuvanted Hla_H35L_ (10 μg/dose) by intracardiac puncture and serum was prepared using serum separator tubes (BD). Serum was pooled within groups, and adoptive transfer of 400 μl of serum was performed by retroorbital injection on the 2 days prior to infection (200 μl/day). For adoptive transfer of Hla-specific antiserum, mice received 100 μl of serum on the day prior to infection. IgG was purified from protective serum using protein A/G columns (Pierce).

### Quantification of antibody levels

Antibody levels were quantified by ELISA, as previously reported (5). 96 well plates were coated with antigen (5 μg), followed by incubation with diluted mouse serum. Detection was performed using alkaline phosphatase (AP)-conjugated goat anti-mouse IgG (1:5000; Jackson ImmunoResearch), and absorbance measured using a GENios spectrophotometer (Tecan).

### Quantification of T cell responses by ELISpot

To quantify T cell responses by ELISpot (5), splenocytes were harvested and aliquoted (5 x 10^5^/well) into 96 well ELISpot plates coated with anti-IL-17 or IFNγ (BD Biosciences). Splenocytes were incubated with heat-killed *S. aureus* (5 x 10^5^ CFU) or purified proteins (1 μM) and incubated at 37°C, 5% CO_2_ for 24 hrs. Biotinylated anti-IFNγ and anti-IL17A detection antibodies (BD Biosciences) and HRP-conjugated anti-biotin (eBioscience) were used as the primary and secondary antibodies, respectively. The plates were washed and substrate solution was added (BD Biosciences); the reaction was terminated and the plates were read using an ImmunoSpot series 1 analyzer (Cellular Technology).

### Quantification of T cell responses by flow cytometry

For quantification of LukE-specific T cell responses, phycoerythrin (PE)-conjugated I-E^d^ LukE_296-311_ class II pMHC tetramers were synthesized by the NIH Tetramer Core Facility (6). Lymphocytes were isolated from spleens and draining lymph nodes and processed into single cell suspensions, then blocked with anti-CD16/32 Ab (clone 2.4G2, Bio X Cell), followed by incubation for 60 min with tetramers at RT. Tetramer enrichment procedures were performed as described previously (6). Briefly, following tetramer staining, tetramer-bound cells were enriched using anti-PE magnetic beads and columns (Miltenyi Biotec). The enriched fractions were collected and used for flow cytometric analysis. Cells were stained for flow cytometry using AquaFluor LiveDead (Life Technologies) solution to exclude dead cells, and a cocktail of dump antibodies was used to exclude unwanted cells: DX5, CD11b (M1/70, BioLegend), F4/80 (BM8), CD19 (1D3), and TER119. Additional antibodies against CD3 (145-2C11), CD4 (RM4-5), CD8 (53-6.7), CD44 (IM7) were used to stain T cells. Antibodies were purchased from eBiosciences, unless specified.

For bulk T cell responses, single-cell suspensions were prepared from lungs, draining lymph nodes, or spleens 7 days after infection. For lung samples, DPBS was injected into the right ventricle following sacrifice in order to remove blood-derived immune cells. Cells were pre-stained with 2.4G2 antibody and LIVE/DEAD™ Fixable Violet Dead Cell Stain Kit (Thermo Fisher Scientific, Cat# L34964) at room temperature for 5 min to prevent unspecific binding and exclude dead cells. For surface staining, cells were incubated with anti-CD90.2 (53-2.1), anti-CD4 (RM4-5), anti-CD8 (53-6.7), anti-CD44(IM7), anti-CXCR5 (L138D7), anti-CD49b (DX5), anti-CD11b (M1/70), anti-F4/80 (BM8), anti-CD19(1D3), and anti-TER119 (TER-119) in the dark at 4°C for 30 min. Following two subsequent washes with stain buffer (PBS +2% FCS), cells were fixed and permeabilized using the Foxp3/Transcription Factor Staining Buffer Set (eBioscience) for 30 min in the dark at room temperature. Cells were then washed twice with Permeabilization Wash Buffer, and incubated with anti- Foxp3 (FJK-16s), anti-RORγt (AFKJS-9) and anti-T-Bet (4B10) overnight in the dark at 4°C. All antibodies were purchased from eBioscience, BioLegend, or BD. Flow cytometry was performed on an LSRII and analyzed with FlowJo software.

### RNA isolation and qRT-PCR

RNA was extracted from lungs stabilized in RNAlater (ThermoFisher) using the RNeasy^®^ Mini Kit (Qiagen). RNA quantity and quality were assessed using a 2100 Bioanalyzer (Agilent). RNA (1 µg) was reverse transcribed using the high-capacity cDNA archive kit (ThermoFisher). qPCR of cytokine and chemokine gene expression was performed using a customized plate (SABioscience) with an ABI Prism 7500 Series Real-time PCR Thermocycler (ThermoFisher). Relative gene expression in the lungs was first normalized against the cycle threshold (Ct) value for the internal control *ldha* and then compared with lungs from control mice using the ΔΔCt method.

### Data analysis

Data were compared using student’s T test, one-way ANOVA with Tukey’s post-test, or log rank (Mantel-Cox) test where appropriate. Differences were considered significant when *p* values were <0.05. For cell numbers, values were log_10_-transformed prior to analysis. For qRT-PCR analysis, gene expression was expressed as fold changes relative to sham (PBS) infected controls and log_10_ transformed for analysis by the Holm-Sidak method for multiple comparisons with α of 0.05; *p*<0.001 was considered significant. All data analysis was performed using GraphPad Prism.

### Study approval

All animal experiments were approved by the Institutional Animal Care and Use Committees at the University of Chicago or the Abigail Wexner Research Institute at Nationwide Children’s Hospital.

## Results

### S. aureus SSTI, but not pneumonia, elicits antibody-mediated protection against secondary SSTI and pneumonia

**Figure 1:**
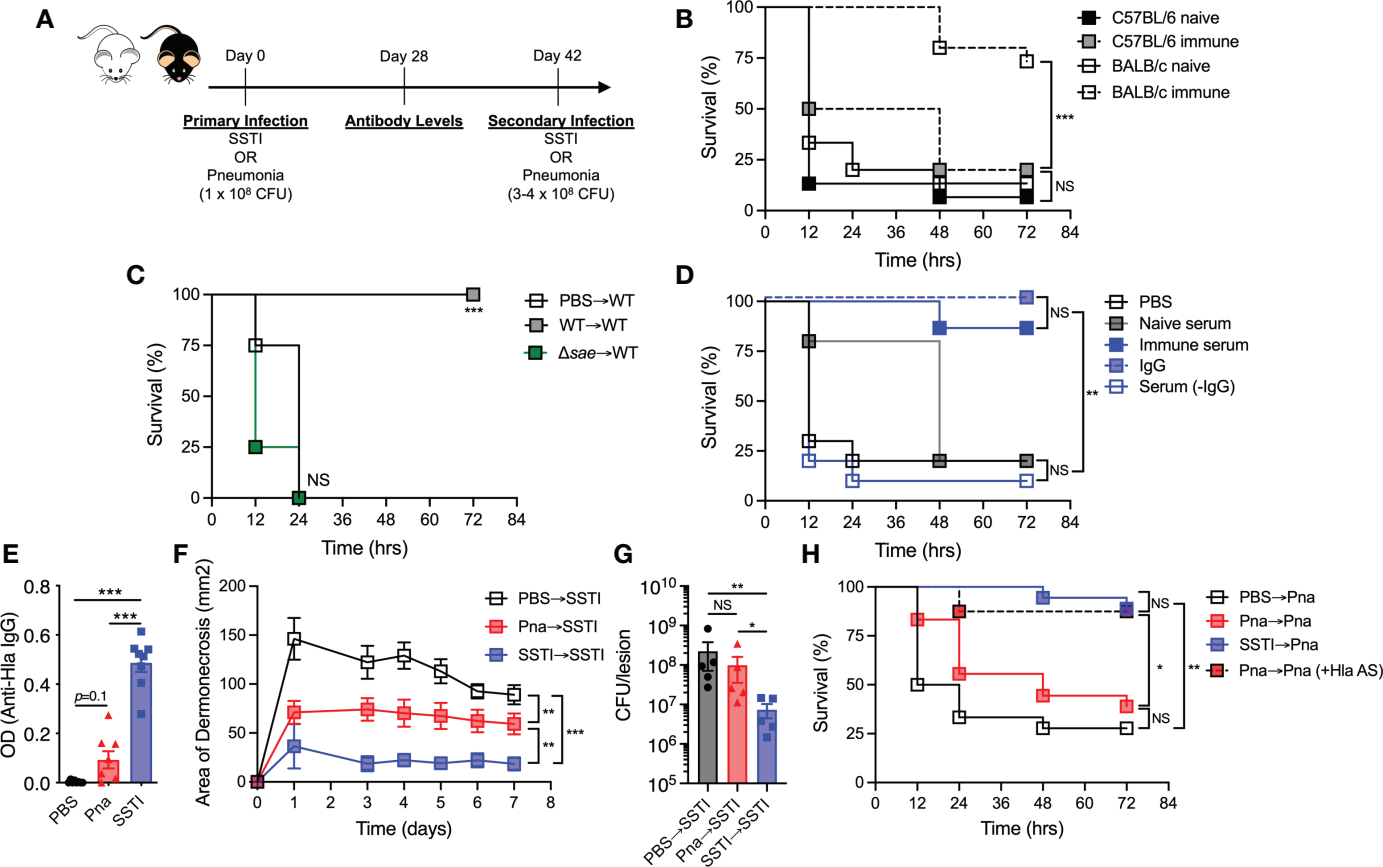
*S. aureus* SSTI, but not pneumonia, elicits antibody-mediated protection against secondary SSTI and pneumonia. **(A)** Model of recurrent SSTI and pneumonia. On day 0, mice were infected with *S. aureus* skin infection (SSTI) or pneumonia (sublethal inoculum, 1 x 10^8^ CFU), followed by quantification of antibody levels 4 weeks later and reinfection with SSTI or high-dose pneumonia (3-4 x 10^8^ CFU) 6 weeks later. **(B)** SSTI protected BALB/c mice, but not C57BL/6 mice, against secondary pneumonia. **(C)** Expression of *saeRS* was necessary to elicit protection against secondary pneumonia in BALB/c mice. **(D)** Passive transfer of serum from previously infected BALB/c mice (“immune”) was sufficient to protect against pneumonia. Purified IgG from immune serum protected against pneumonia, but serum from which IgG was removed (Serum (-IgG)) did not. **(E)** There were increased levels of anti-Hla IgG in BALB/c mice following SSTI (4 weeks post-infection), compared with pneumonia. **(F)** Primary SSTI elicited stronger protection against secondary dermonecrosis, compared with primary pneumonia. **(G)** Primary SSTI, but not primary pneumonia, resulted in enhanced clearance of bacteria from skin lesions. **(H)** Primary SSTI, but not primary pneumonia, protected against secondary pneumonia. Passive transfer of Hla-specific antiserum (AS) rescued protection in mice that received primary pneumonia. **(F, G)** N=5 mice/group from one representative experiment (of 2 performed). **(B–E, H)** N=8-15 mice/group pooled from 2-3 independent experiments; all data are presented as mean ± SEM. Data were compared using log-rank (Mantel-Cox) test **(B–D, H)**, one-way ANOVA with Tukey’s post-test **(E, G)**, or two-way ANOVA with repeated measures **(F)**. * indicates *p*<0.05, ** *p*<0.01, *** *p*<0.001, NS indicates not significant.

We previously demonstrated that *S. aureus* SSTI elicits protective immunity against secondary SSTI in BALB/c mice, but not C57BL/6 mice, by eliciting antibody and T cell responses specific for a subset of antigens regulated by the *saeRS* regulatory operon (5, 9). To test whether SSTI could also protect against a more invasive infection, we modified our model of recurrent *S. aureus* infection, with the primary SSTI followed 6 weeks later by secondary pneumonia (model, **Figure 1A**). We observed that while SSTI did not protect C57BL/6 mice against mortality during secondary pneumonia, BALB/c mice were strongly protected (**Figure 1B**). As we observed with recurrent SSTI (9), primary SSTI with an isogenic *saeRS* deletion mutant (*Δsae*) failed to elicit protection against secondary pneumonia with the wild-type isolate in BALB/c mice (**Figure 1C**), suggesting that the mechanisms of protective immunity against pneumonia are similar to those that protect against SSTI in that *saeRS* expression during primary infection is necessary for protection.

Because antibody-mediated immunity is important for protection against recurrent SSTI (5), we tested whether antibody could mediate protection against secondary pneumonia by passive transfer of “immune” serum from previously infected BALB/c mice (4 weeks post-secondary SSTI) into naïve mice prior to pneumonia. Compared with mice that received either PBS or serum from naïve mice, recipients of immune serum were protected against pneumonia (**Figure 1D**), demonstrating that, similar to the recurrent SSTI model, antibodies are sufficient to mediate protection against secondary pneumonia. Protection was specifically mediated by antibody, because purified IgG, but not serum from which IgG was removed, conferred protection against pneumonia (**Figure 1D**). We hypothesized that a more invasive infection, such as pneumonia, would elicit higher levels of antibody, compared with the relatively superficial SSTI. To test this, BALB/c mice received either SSTI or a low inoculum of *S. aureus* (1 x 10^8^ CFU) that results in sublethal pneumonia, followed by quantification of antibody levels against the *sae*-regulated α-hemolysin (Hla) (model, **Figure 1A**). Surprisingly, SSTI elicited significantly higher levels of antibody against Hla, compared with pneumonia (**Figure 1E**, **Figure S1**). The levels of anti-Hla IgG elicited by primary SSTI, while higher than pneumonia, were not as high as those in “protective” serum (post secondary SSTI) used for passive transfer (**Figures 1E**, **Figure S1**). There was a trend toward higher anti-Hla IgG levels following pneumonia, compared with PBS, but the differences were not significant (**Figure 1E**, **Figure S1**). There were no significant differences in the levels of IgG against LukE and LukS-PV (data not shown).

Hla-specific antibodies protect against SSTI and pneumonia in mice (10); therefore, our findings suggested that primary pneumonia would not elicit protection against SSTI or secondary pneumonia. To test this, primary sublethal pneumonia or SSTI was followed 6 weeks later by secondary SSTI (model, **Figure 1A**). Consistent with lower levels of elicited Hla-specific IgG, primary pneumonia resulted in larger skin lesions during secondary SSTI, compared with primary SSTI (**Figure 1F**). There was, however, modest protection elicited by pneumonia, consistent with the trend toward increased anti-Hla antibody levels (**Figures 1E, F**, **Figure S1**). Similarly, bacterial clearance from the skin lesions was less effective during SSTI primed by pneumonia, compared with priming by SSTI (**Figure 1G**). We next tested whether primary pneumonia would protect against secondary pneumonia with a higher inoculum (3-4 x 10^8^ CFU). Indeed, primary pneumonia failed to protect against secondary pneumonia (**Figure 1H**), a phenotype that was rescued by passive transfer of Hla-specific antiserum prior to secondary infection. Thus, contrary to our hypothesis, these findings demonstrate that protective immunity elicited by SSTI, even against an invasive infection such as pneumonia, is superior to immunity elicited by sublethal pneumonia. Our findings also underscore the protective role for anti-Hla antibodies.

### SSTI, but not pneumonia, elicits antigen-specific memory T cell responses

**Figure 2:**
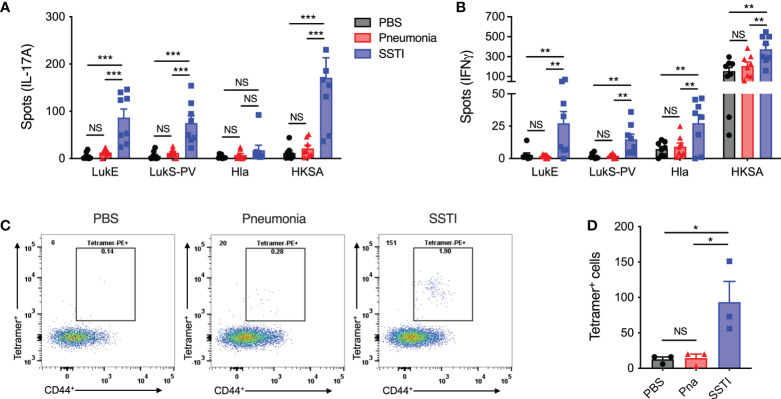
SSTI, but not pneumonia, elicits antigen-specific memory T cells. 4 weeks following primary SSTI or pneumonia, *S. aureus*-specific T cell responses were quantified by ELISpot or pMHC tetramers specific for a common LukE/LukS-PV-specific epitope (LkT/C:I-E^d^). **(A, B)** Incubation of splenocytes with heat-killed *S. aureus* (HKSA) resulted in more IL-17A- and IFNγ-secreting T cells following SSTI, compared with pneumonia. There were also more cytokine-secreting T cells following incubation with LukE (IL-17A, IFNγ), LukS-PV (IL-17A, IFNγ), and Hla (IFNγ) in mice that received SSTI, compared with mice that received pneumonia. **(C, D)** There were increased numbers of pMHC (LkT/C:I-E^d^) staining CD4^+^ CD44^+^ T cells following SSTI, but not pneumonia. N=8 mice/group pooled from 2 independent experiments **(A, B)** or 3 mice/group from one representative experiment of 2 repeats **(D)**. All data are presented as mean ± SEM. Data were compared using one-way ANOVA with Tukey’s post-test. * indicates *p*<0.05, ** *p*<0.01, *** *p*<0.001, NS indicates not significant.

We hypothesized that the lower levels of anti-Hla IgG would be reflected in impaired generation of *S. aureus*-specific memory T cells. Therefore, we compared *S. aureus* antigen-specific memory T cell responses 4 weeks after primary SSTI or pneumonia. To accomplish this, we first used an ELISpot assay to quantify *S. aureus*-specific IL-17A and IFNγ T cell responses in whole-spleen homogenates of convalescent mice. Cytokine production was stimulated by culturing splenocytes with heat-killed *S. aureus* (HKSA). Similar to the antibody responses we observed, there were higher numbers of *S. aureus*-specific IL-17A and IFNγ-secreting T cells following SSTI, compared with pneumonia, at 4 weeks post-infection (**Figures 2A, B**).

We further quantified IL-17A and IFNγ responses against Hla and the *sae*-regulated leukotoxins LukE and LukS-PV. Consistent with our previous report (6), we observed that SSTI elicited IL-17A and IFNγ-secreting cells following culture with LukE and LukS-PV and IFNγ-secreting cells following culture with Hla (**Figures 2A, B**). In contrast, pneumonia did not result in increased accumulation of IL-17A and IFNγ-producing T cells specific for Hla, LukE, or LukS-PV in the spleen (**Figures 2A, B**). Collectively, these observations demonstrate that SSTI, despite being a less invasive infection, was able to elicit more robust systemic *S. aureus*-specific Th17 and Th1 memory responses, compared with the more invasive pneumonia.

To address the potential limitation that the ELISpot assay only quantified memory T cell responses based on IL-17A and IFNγ production, we used a previously characterized peptide MHC tetramer specific for a conserved immunodominant leukotoxin C-terminal epitope, called LkT/C (6). The LkT/C:I-E^d^ pMHC tetramer allowed us to quantify all LukE/LukS-PV-specific T cells pooled from the spleen and dLNs 4 weeks following SSTI or pneumonia. Using this approach, we found significantly more LkT/C^+^ CD44^+^ CD4^+^ T cells in mice that recovered from SSTI, compared with those that recovered from pneumonia (**Figures 2C, D**), thus confirming that only SSTI elicited *S. aureus*-specific memory CD4^+^ T cells. Unfortunately, we have not been able to develop Hla-specific pMHC tetramers to track endogenous CD4^+^ T cells, so we were not able to confirm whether SSTI also elicited higher numbers of Hla-specific memory T cells.

### SSTI and pneumonia elicit local, but not systemic, T cell expansion

**Figure 3:**
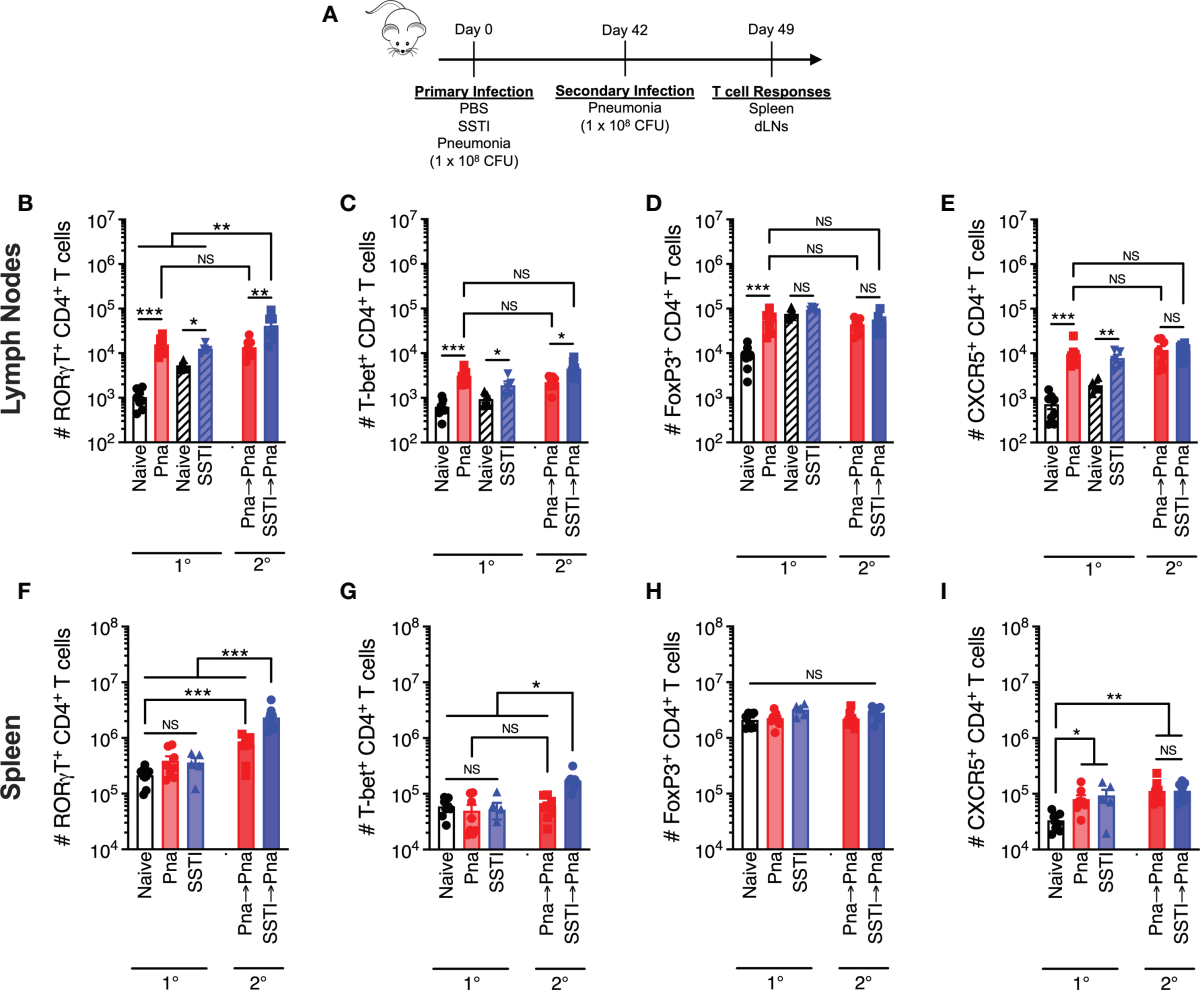
Local and systemic T cell responses against *S. aureus* pneumonia and SSTI. CD4^+^ T cell populations were quantified 7 days following primary SSTI or pneumonia (Pna) or secondary pneumonia following SSTI (SSTI→pna) or pneumonia (pna→pna) [model in **(A)**] in the draining lymph nodes (dLNs), **(B–E)** or the spleen **(F–I)**. **(B–E)** There were increased numbers of RORγt^+^, T-bet^+^, FoxP3^+^, and CXCR5^+^ CD4^+^ T cells in the mediastinal dLNs (open bars) following pneumonia, compared with naïve mice, and increased numbers of RORγt^+^, T-bet^+^, and CXCR5^+^ CD4^+^ T cells in the inguinal dLNs (hatched bars) following SSTI. Primary SSTI primed greater expansion of RORγt^+^ and T-bet^+^ CD4^+^ T cells in the mediastinal dLNs following secondary pneumonia, compared with those primed by primary pneumonia. **(F–I)** There was no increase in RORγt^+^, T-bet^+^, or FoxP3^+^ CD4^+^ T cell populations in the spleen following pneumonia or SSTI, but there were increased numbers of CXCR5^+^ CD4^+^ T cells following pneumonia and SSTI, compared with naïve mice. Primary SSTI primed greater expansion of RORγt^+^ and T-bet^+^ CD4^+^ T cells in the spleen following secondary pneumonia, compared with those primed by primary pneumonia. N=5-8 mice group pooled from 2 independent experiments; all data are presented as mean ± SEM. Data were compared using one-way ANOVA on log_10_-transformed values with Tukey’s post-test. * indicates *p*<0.05, ** *p*<0.01, *** *p*<0.001, NS indicates not significant.

We hypothesized that pneumonia failed to elicit protective antibody and T cell responses due to blunted local T cell responses during primary infection. To test this, bulk T cell responses following primary SSTI or sublethal pneumonia in the draining lymph nodes (dLNs; pneumonia – mediastinal, SSTI – inguinal, hatched bars) on day 7 after infection were quantified by flow cytometry for the following CD4^+^ T cell subsets: Th17 (RORγt^+^), Th1 (T-bet^+^), regulatory T cells (Treg; FoxP3^+^), and follicular helper T cells (Tfh; CXCR5^+^)(model, **Figure 3A**, gating strategy **Figure S2**). Following primary pneumonia, there was an increase in the numbers of Th17, Th1, Treg, and Tfh CD4^+^ T cells in the mediastinal dLNs (**Figures 3B–E** 1°). There were also increased numbers of Th17, Th1, and Tfh CD4^+^ cells in the inguinal dLNs following SSTI (**Figures 3B, C, E** 1°), but not Tregs (**Figure 3D** 1°). The magnitude of increase in CD4^+^ T cell subsets in the mediastinal LNs following pneumonia was consistently larger than the increase in inguinal LNs following SSTI, suggesting that, contrary to our hypothesis, pneumonia effectively expands local effector T cells.

We next investigated systemic responses by quantifying Th1, Th17, Treg, and Tfh CD4^+^ T cells in the spleen 7 days after primary pneumonia or SSTI. In contrast to the dLNs, we observed no significant expansion of Th17, Th1, or Treg subsets in the spleen following pneumonia or SSTI (**Figures 3F–H** 1°). There was expansion of Tfh cells in the spleen following SSTI and pneumonia, compared with naïve mice, but there were no significant differences between pneumonia or SSTI (**Figure 3I** 1°). Together, these results demonstrate that SSTI and pneumonia each resulted in expansion of local effector CD4^+^ T cells, possibly even moreso following pneumonia. In contrast, expansion of systemic bulk CD4^+^ T cells following either infection was considerably more modest, possibly reflecting the early time point studied.

### SSTI primes greater expansion of T cells following secondary pneumonia

Our findings that SSTI elicited higher numbers of *S. aureus*-specific Th17 and Th1 memory cells, compared with pneumonia, led us to compare the magnitude of the recall T cell responses upon challenge with secondary pneumonia. We quantified T cells in the mediastinal LNs and spleens of mice 7 days after infection with secondary pneumonia following primary pneumonia (Pna→Pna) or secondary pneumonia following primary SSTI (SSTI→Pna) and compared these recall responses with those elicited following primary pneumonia (Pna)(model in **Figure 3A**). Consistent with our hypothesis, there were more RORγt^+^ and T-bet^+^ CD4^+^ T cells in the mediastinal LNs of the SSTI→Pna group, compared with the Pna or Pna→Pna groups, suggesting that SSTI primed greater expansion of Th17 and Th1 cells in the dLNs following secondary pneumonia than did primary pneumonia (**Figures 3B, C** 2°). However, there were no significant differences in the number of FoxP3^+^ or CXCR5^+^ T cells in the dLNs (**Figures 3D, E** 2°) among the groups (Pna, Pna→Pna, SSTI→Pna). Similarly, there were more RORγt^+^ and T-bet^+^ CD4^+^ T cells in the spleen of the SSTI→Pna group, compared with the Pna or Pna→Pna groups (**Figures 3F, G** 2°), suggesting that SSTI also primed greater expansion of systemic Th17 and Th1 cells. As we observed in the dLNs, there were no significant differences in the number of FoxP3^+^ or CXCR5^+^ T cells in the spleen (**Figures 3H, I** 2°) among the groups (Pna, Pna→Pna, SSTI→Pna). Taken together, these results demonstrate that, despite eliciting expansion of local CD4^+^ T cells following primary infection, pneumonia fails to prime expansion of memory Th17 and Th1 cells in the dLNs, and to a lesser extent than does SSTI in the spleen, upon recall by secondary pneumonia.

### SSTI primes greater expansion of T cells in the lung following secondary pneumonia

**Figure 4:**
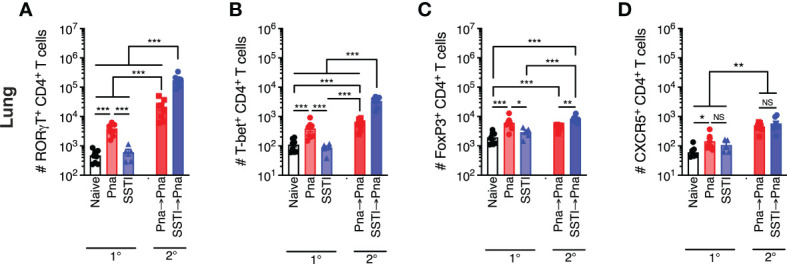
T cell responses in the lung following *S. aureus* pneumonia and SSTI. Using the model depicted in **Figure 3A**, CD4^+^ T cell subsets were quantified in the lungs 7 days following primary SSTI or pneumonia (Pna) or secondary pneumonia following SSTI (SSTI→pna) or pneumonia (pna→pna). There were higher numbers of RORγt^+^ **(A)**, T-bet^+^ **(B)**, FoxP3^+^ **(C)**, and CXCR5^+^ **(D)** CD4^+^ T cells in the lungs of mice that received pneumonia, compared with naïve or SSTI groups. Mice that were primed with SSTI prior to secondary pneumonia (SSTI→Pna) had higher numbers of RORγt^+^ **(A)**, T-bet^+^ **(B)**, and FoxP3^+^ **(C)** CD4^+^ T cells in the lungs, compared with mice that received primary pneumonia (Pna) or mice that received secondary pneumonia following priming with primary pneumonia (Pna→Pna). Mice that were primed with SSTI (SSTI→Pna) or pneumonia (Pna→Pna) prior to secondary pneumonia had increased numbers of CXCR5^+^ CD4^+^ T cells, compared with naïve mice or mice that received primary pneumonia (Pna→Pna) **(D)**. N=5-8 mice/group pooled from 2 independent experiments. All data are presented as mean ± SEM. Data were compared using one-way ANOVA on log_10_-transformed values with Tukey’s post-test. * indicates *p*<0.05, ** indicates *p*<0.01, *** *p*<0.001. NS means not significant.

We next tested whether a more robust lung-specific recall T cell response would be observed in mice that were primed by pneumonia compared with those primed by SSTI. To test this, we first quantified CD4^+^ T cell subsets in the lung 7 days after primary pneumonia or SSTI (model in **Figure 3A**). As expected, there were more RORγt^+^, T-bet^+^, FoxP3^+^, and CXCR5^+^ CD4^+^ T cells in the lung following primary pneumonia, compared with mice that received SSTI or PBS (**Figures 4A–D** 1°). Next, we compared SSTI- and pneumonia-primed CD4^+^ T cell responses in the lung 7 days after secondary pneumonia. Strikingly, as we observed with responses in the dLNs and spleen, there were more (nearly 10-fold) RORγt^+^ and T-bet^+^ CD4^+^ T cells in lungs of the SSTI→Pna group, compared with the Pna or Pna→Pna groups (**Figures 4A, B** 2°). These findings demonstrated that Th17 and Th1 cells in the lung following secondary pneumonia were expanded in greater numbers when primed by SSTI, compared with priming by pneumonia. This was an unexpected finding because primary pneumonia elicited higher numbers of Th17 and Th1 cells in the lung, compared with SSTI, and raised the possibility that Tregs may have been preferentially elicited by Pna, which then modulated the recall T cell response. However, as we observed with Th17 and Th1 responses, there were more FoxP3^+^ CD4^+^ T cells in the SSTI→Pna group, compared with the Pna→Pna group (**Figure 4C** 2°). Collectively, these data suggest that Treg expansion by pneumonia was unlikely the cause of reduced recall Th17 and Th1 responses. Finally, CXCR5^+^ CD4^+^ T cells were expanded in similar numbers in the Pna→Pna and SSTI→Pna groups, and each was significantly higher than the Pna group (**Figure 4D** 2°). Taken together, these results demonstrate that the relative magnitude of expansion of systemic, local and lung effector T cells elicited by primary SSTI or pneumonia do not predict the magnitude of recall T cell expansion during, or protection against, secondary pneumonia.

### Priming by SSTI permits early inflammation and T cell expansion in the lungs during secondary pneumonia

**Figure 5:**
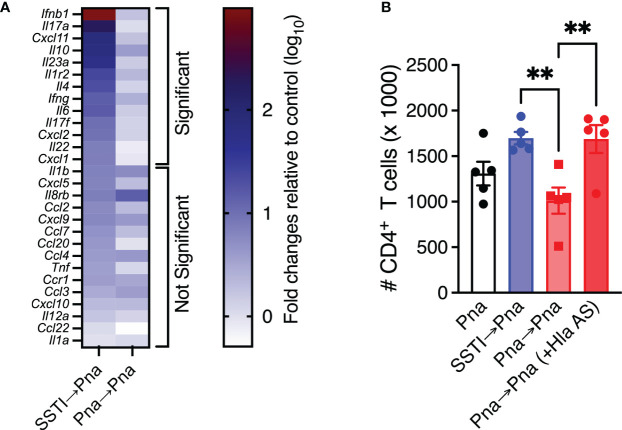
Priming by SSTI permits early inflammation and T cell expansion in the lungs during secondary pneumonia. **(A)** Heat map of inflammatory gene expression quantified by qRT-PCR on lung homogenates 42 hours following secondary pneumonia. Results are expressed as fold differences (log_10_ transformed) compared with sham-infected mice using the ΔΔCt method. Mice that were primed with SSTI prior to secondary pneumonia (SSTI→Pna) had significantly higher expression of 13 inflammatory genes, including chemokines and cytokines, compared with those that were primed with pneumonia (Pna→Pna). There were no significant differences in the expression of 15 inflammatory genes. **(B)** Passive transfer of Hla-specific antiserum (AS) prior to secondary pneumonia rescued CD4^+^ T cell numbers in the lungs early (42 hours) following inoculation. N=5 mice/group from one representative experiment; each experiment was performed at least twice. All data are presented as mean ± SEM. Data were compared using the Holm-Sidak method for multiple comparisons **(A)** or one-way ANOVA on log_10_-transformed values with Tukey’s post-test **(B)**. ** indicates *p*<0.01, NS indicates not significant.

We recently reported that Hla-specific antibody-mediated protection against dermonecrosis is characterized by enhanced early inflammation in the skin (11). To test if protection in this model is also associated with enhanced early local inflammatory responses, mice were sacrificed 42 hrs after secondary pneumonia (Pna→Pna or SSTI→Pna) and RNA was isolated and purified from lung homogenates. qRT-PCR was performed to quantify inflammatory gene transcripts and expression was quantified as fold-changes relative to the lungs of healthy control mice. Based on our previous report of inflammatory genes whose expression was elevated following *S. aureus* pneumonia, we quantified transcripts for 28 genes (12). Of these, there were significantly higher levels of 13 inflammatory gene transcripts in the lungs of mice from the SSTI→Pna group, compared with the Pna→Pna group (**Figure 5A**), while there were 15 inflammatory gene transcripts whose levels were not significantly different between the 2 groups. The transcripts for which there were increased expression included genes encoding chemokines (*cxcl1*, *cxcl2*, *cxcl11*), interleukins (*il4*, *il6*, *il10, il17a, il17f, il22, il23a*), and interferons (*ifnb1, ifng*). These findings support the hypothesis that protection against secondary pneumonia elicited by SSTI is accompanied by increased local inflammatory responses in the lung during secondary pneumonia.

We also previously reported that Hla-specific antibody-mediated protection against dermonecrosis was due to protection of immune cell populations by Hla toxin neutralization (11). Since anti-Hla IgG levels were lower following primary pneumonia compared with SSTI and passive transfer of Hla-specific antiserum prior to secondary infection restored protection in pneumonia-primed mice, we next tested whether the higher levels of anti-Hla antibodies elicited by SSTI were responsible for the higher numbers of T cells we observed in the lungs the SSTI→Pna group, compared with the Pna→Pna group. To test this, we quantified T cells in the lung early after infection (42 hrs) following Pna, Pna→Pna or SSTI→Pna. A subset of the Pna→Pna group received passive transfer of Hla-specific antiserum prior to secondary pneumonia to test the role of antibody-mediated Hla neutralization. Consistent with this hypothesis, transfer of Hla-specific antiserum to the Pna→Pna group “rescued” CD4^+^ T cells to numbers comparable with the SSTI→Pna group (**Figure 5B**). There were, however, no significant differences in CD8^+^ or γδ T cells (**Figure S3**). Taken together, these results suggest that SSTI primes enhanced inflammation in the lungs early after secondary pneumonia and that neutralization of Hla enabled expansion of CD4^+^ T cell populations. However, it remains unclear if these two processes are interdependent or merely associated with one another.

## Discussion

In a mouse model of recurrent *S. aureus* infection, we found that SSTI elicited protective immunity against secondary pneumonia. Contrary to our hypothesis, pneumonia failed to elicit protection against secondary SSTI and pneumonia. Despite the differences in protection, SSTI and pneumonia each elicited early local expansion of effector CD4^+^ T cell subsets (day 7 post-infection). However, only SSTI elicited high levels of antibody against Hla and memory T cell responses against LukE and LukS-PV, providing a potential explanation for the differential protection against subsequent infection. Surprisingly, SSTI also primed greater expansion of T cells in the draining lymph nodes and lungs following secondary pneumonia. This phenotype may be explained by toxin expression during secondary infection coupled with the inability to generate anti-Hla IgG responses. During secondary pneumonia, Hla-mediated necrosis in the lung is likely neutralized by the high levels of anti-Hla antibody elicited only by SSTI. Taken together, these findings emphasize the importance of understanding protective antibody and memory T cells in the context of the site of infection.

These results are consistent with our model of recurrent SSTI, in which primary SSTI protected BALB/c mice against secondary SSTI in a manner that was dependent on expression of the regulatory operon *saeRS* during the primary infection (5, 9). Therefore, expression of *sae*-regulated toxins drives the pathogenesis of *S. aureus* SSTI and pneumonia (8), but is also necessary to elicit protective immunity in the context of SSTI. Because Hla is the major virulence factor that determines the severity of *S. aureus* pneumonia in mice (13) and vaccination against Hla is protective (10), our findings of high anti-Hla IgG levels following *S. aureus* SSTI, but not pneumonia, support that antibody-mediated immunity against Hla is critical for protection against secondary *S. aureus* pneumonia. Notably, while anti-Hla IgG was not elicited by *S. aureus* pneumonia in BALB/c mice, antibody levels against LukE and LukS-PV were comparable with SSTI infection. These findings are consistent with our previous observations that SSTI did not elicit an anti-Hla IgG response or protection against secondary pneumonia in C57BL/6 mice but were able to generate an IgG response to LukE and LukS-PV. In those studies, we showed that SSTI did not elicit antibody responses against Hla or T cell responses against LukE and LukS-PV due to an inability of peptides from these antigens to be presented on C57BL/6 MHC II^b^ (6, 9). It should be noted that the two component toxin Panton-Valentine leucocidin (comprised of LukF-PV and LukS-PV) is not able to bind its cellular receptor, C5aR, in mice (14); therefore, conclusions on the role of LukS-PV is pathogenesis and elicited protective immunity in mouse models are difficult to ascertain. Whether similar mechanisms explain the lack of anti-Hla IgG responses in BALB/c during *S. aureus* pneumonia requires further investigation.

Consistent with our previous reports using the SSTI model (5, 6), generation of protective antibody was accompanied by expansion of antigen-specific memory T cells. While the numbers of bulk effector T cells were comparable on day 7 post-pneumonia or SSTI, *S. aureus*-specific memory T cells were significantly fewer at week 4 post-pneumonia compared with SSTI. One potential explanation for this finding could be that the rapid clearance of bacteria from the lungs of mice after pneumonia (3-5 days), compared with sustained bacterial persistence after SSTI (at least 2 weeks), may preclude the development of Hla-specific antibody and sustained memory T cell responses. This explanation could reconcile our findings of expansion of bulk effector T cells in the dLNs and lungs but not of antigen-specific memory T cells following pneumonia. Consistent with this idea, sustained antigen persistence is necessary for antigen-specific memory T cell responses in mouse models of *Salmonella* and *Chlamydia* infection (15, 16). Other possible explanations may include tissue-specific defects in antigen presentation or impairment of Tfh expansion and subsequent anti-Hla IgG development. It should also be noted that we cannot fully exclude a role for trained immunity in contributing to protection in our model. Future studies will test these possibilities.

A protective role for effector T cells in our model is suggested by the observation that impaired expansion of recall T cells was observed upon secondary infection with *S. aureus* pneumonia in mice that had experienced prior pneumonia compared with those that received SSTI. We speculate that in the face of a large bolus of *S. aureus* in the lungs following intranasal inoculation, accumulation of toxins such as Hla results in the depletion of antigen-presenting and T cells in the lung and increased tissue necrosis. If this hypothesis is true, one would anticipate that neutralization of Hla would rescue this phenotype. Indeed, protective immunity was associated with higher levels of inflammatory gene transcripts and adoptive transfer of Hla-specific anti-serum resulted in more CD4^+^ T cells in the lung and protection against lethal pneumonia. This observation is consistent with several reports that *S. aureus* toxins inhibit adaptive immune responses *via* direct toxicity toward antigen-presenting cells and T cells (7, 17, 18). We reported a similar phenomenon in *S. aureus* dermonecrosis (11). However, it is important to consider that lethal pneumonia is an “all-or-nothing” phenotype driven by toxin expression in the lung disrupting the alveolar epithelium (19) – this complicates our ability to understand the subtle changes in local and systemic immune responses that may modify the response to infection. Together, our findings suggest that tissue-specific toxin expression can either evade or drive the development of protective immunity, depending on the ability to generate anti-toxin neutralizing antibodies. Future work will test this hypothesis. Moreover, whether effector T cells directly contribute to protection against secondary pneumonia is not yet known.

Consistent with animal studies, the host transcriptional response in children with *S. aureus* infection depends on the site of infection and the extent of dissemination, with stronger transcriptional activation of multiple immune pathways following pneumonia, compared with localized or osteoarticular infection (20). One possible explanation to reconcile these seemingly discrepant findings is that invasive *S. aureus* infection in children results in strong innate monocyte-dependent responses but decreased central memory T cell responses (21). Therefore, it is tempting to speculate that invasive infections, despite eliciting local inflammation, may not expand antigen-specific memory T cells or may even suppress adaptive responses *via* toxin expression. If this is the case, it is also possible that this phenotype may be specific to younger children prior to the development of toxin-specific antibodies. Conversely, localized infections may elicit greater expansion of memory T cells in the absence of intense innate immune responses, at least for some individuals.

Although direct comparison of elicited adaptive responses following SSTI and pneumonia has not been thoroughly documented, several clinical reports suggest that more invasive infections may elicit higher antibody levels. For example, children with invasive *S. aureus* infections had higher convalescent levels of antibodies against Hla and PVL, compared with those with less severe infections, despite having similar or lower levels at the onset of illness (3, 22). Importantly, higher antibody levels correlated with a lower rate of recurrent infection in children (3). However, these studies are limited by a small number of patients with pneumonia. There is also some evidence that noninvasive infections, such as SSTI, elicit protective immunity against pneumonia. For example, patients with a prior history of SSTI had improved outcomes in necrotizing pneumonia, with lower severity of illness and lower mortality (23, 24). A systematic review also identified the absence of an associated SSTI as a risk factor for mortality during pneumonia (25). Future studies will be needed to directly compare responses elicited by different infectious syndromes and determine whether memory T cells elicited by infection reflect antibody levels.

In summary, we found that *S. aureus* SSTI, but not pneumonia, elicits protective immunity against secondary pneumonia by virtue of eliciting higher levels of Hla-specific antibody. Protection was also associated with expansion of *S. aureus*-specific memory T cells. Taken together, these findings highlight the importance of understanding protective systemic immunity in the context of tissue-specific elicited immune responses.

## Data availability statement

The raw data supporting the conclusions of this article will be made available by the authors, without undue reservation.

## Ethics statement

The animal study was reviewed and approved by Animal Care and Use Committees at the University of Chicago and Nationwide Children’s Hospital.

## Author contributions

PB designed the studies, conducted experiments, analyzed data, and wrote the manuscript. YS designed the studies, conducted experiments, analyzed data, and wrote the manuscript. CY designed the studies, conducted experiments, and analyzed data. ZL conducted experiments and analyzed data. FZ conducted experiments and analyzed data. AC designed the studies and wrote the manuscript. CM designed the studies, analyzed data, and wrote the manuscript. PB and YS contributed equally; PB is listed first due to a greater role in designing the studies and writing the manuscript. All authors contributed to the article and approved the submitted version.

## Funding

This work was supported by the National Institute for Allergy and Infectious Diseases (AI125489 to CM) and the Abigail Wexner Research Institute at Nationwide Children’s Hospital.

## Acknowledgments

The plasmid for purification of HlaH35L was a gift from Dr. Juliane Bubeck Wardenburg (Washington University St. Louis). pMHC tetramers were synthesized by the NIH Tetramer Core Facility.

## Conflict of interest

The authors declare that the research was conducted in the absence of any commercial or financial relationships that could be construed as a potential conflict of interest.

## Publisher’s note

All claims expressed in this article are solely those of the authors and do not necessarily represent those of their affiliated organizations, or those of the publisher, the editors and the reviewers. Any product that may be evaluated in this article, or claim that may be made by its manufacturer, is not guaranteed or endorsed by the publisher.
