# Supplementary material for: Tissue specificity drives protective immunity against Staphylococcus aureus infection
Source: Front Immunol. 2022 Aug 2;13:795792. doi: 10.3389/fimmu.2022.795792 (PMC9380724; doi:10.3389/fimmu.2022.795792)
Supplement: Supplementary file 1 [file DataSheet_1.docx]

**SUPPLEMENTARY FIGURES**

**Figure S1. Anti-Hla IgG titers following SSTI and pneumonia.** Serum was diluted serially (2-fold) followed by Hla IgG-specific ELISA. Antibody levels following primary SSTI or pneumonia (pna) were compared with “protective” serum following secondary SSTI. All data are presented as mean ± SEM (N=4-5 samples/group except protective serum which was a pooled sample). Data were compared using two-way ANOVA with Tukey’s post-test. ** indicates *p*<0.01, NS indicates not significant.

**Figure S2. Gating strategy for flow cytometry studies.** Cells from dLNs, spleen, or lungs were sorted by forward and side scatter followed by live/dead staining. CD3^+^ T cells were identified and gated on CD4^+^ and CD8^+^ T cells, followed by gating of CD4^+^ T cells on RORγt^+^, T-bet^+^, FoxP3^+^, and CXCR5^+^ cells.

**Figure S3. CD8^+^ and γδ T cells in the lung following secondary pneumonia**. Passive transfer of Hla-specific antiserum (AS) prior to secondary pneumonia did not rescue CD8^+^ (A) or γδ (B) T cell numbers in the lungs early (42 hours) following inoculation. N=5 mice/group from one representative experiment; each experiment was performed at least twice. All data are presented as mean ± SEM. Data were compared using one-way ANOVA on log_10_-transformed values with Tukey’s post-test. NS indicates not significant.
